# Cathode coating using LiInO_2_-LiI composite for stable sulfide-based all-solid-state batteries

**DOI:** 10.1038/s41598-019-44629-x

**Published:** 2019-05-30

**Authors:** Hwan Wook Kwak, Yong Joon Park

**Affiliations:** Department of Advanced Materials Engineering, Kyonggi University, 154-42, Gwanggyosan-Ro, Yeongtong-Gu, Suwon-Si, Gyeonggi-Do 16227 Republic of Korea

**Keywords:** Batteries, Batteries

## Abstract

All-solid-state batteries with inorganic solid electrolytes are ideal to overcome the safety issues related to the flammable organic electrolyte in lithium ion batteries. Sulfide materials are promising inorganic electrolytes due to their high ionic conductivity and good elasticity. Nevertheless, their application is limited by their high reactivity and instability at the cathode/electrolyte (Li[Ni_0.8_Co_0.15_Al_0.05_]O_2_/75Li_2_S–22P_2_S_5_–3Li_2_SO_4_) interface. In this study, LiInO_2_ and LiInO_2_–LiI were introduced as new cathode coating materials to suppress such undesirable reactions. The LiInO_2_–LiI composite coating layer reduced the undesirable interfacial reactions and prevented the diffusion of S and P ions from the sulfide electrolyte to the oxide cathode. Moreover, the electrochemical properties of all-solid-state cells were improved by the cathode coating. The LiInO_2_–LiI-coated electrode presented better rate capability and lower impedance than the pristine and LiInO_2_-coated electrodes. Hence, the LiInO_2_–LiI composite coating was successful at improving the cathode stability while providing superior electrochemical properties.

## Introduction

Nowadays, lithium ion batteries (LIBs) are used to power everything from small portable devices to electric vehicles and large scale machinery such as energy storage systems^1–6^. However, they suffer from safety issues arising from the flammability of the organic liquid electrolyte used. Moreover, the consequences become increasingly serious for larger LIBs. Although it is not yet widely known to the public, there have been several fires in electric vehicles and energy storage systems involving LIBs. This could prove fatal for the current market expansion of LIBs. Ultimately, the flammable liquid electrolyte needs to be replaced with a non-flammable solid electrolyte^7–11^. Many superionic conductors have been explored as candidate materials^12–17^. Among them, sulfide superionic conductors have attracted a lot of attention due to their high ionic conductivity^18–20^. Several state-of-the art sulfide-based solid electrolytes such as Li_10_GeP_2_S_12_ (LGPS) and Li_9.54_Si_1.74_P_1.44_S_11.7_Cl_0.3_ (LSiPSCl) have even demonstrated conductivities far greater than those of organic liquid electrolytes^12,13^. Furthermore, sulfide materials can form interfacial connections with the cathode through mechanical pathways without the need for high temperature sintering because of their ductility and elasticity. Nevertheless, the commercialization of sulfide-based all-solid-state batteries (ASSBs) has been limited by the high reactivity of sulfides, that causes instability at the sulfide electrolyte/electrode interface. In particular, the interface reactions that occur with oxide cathodes form an undesirable interfacial layer^21–23^ which deteriorates the capacity, rate capability, and cyclic performance of ASSBs.

It has been demonstrated that such interfacial instability can be suppressed by surface modification of the cathode by coating it with a stable oxide, for example LiNbO_3_^24^, Li_4_Ti_5_O_12_^25^, Li_3*x*_La_2/3−*x*_TiO_3_^26^, and Li_2_O–SiO_2_^27^. However, the research on surface modification to enhance interfacial stability in sulfide-based ASSBs is in its infancy compared to the broad literature on reducing the unwanted reactions that arise from organic liquid electrolytes in LIBs. Moreover, the role of the coating layer in ASSBs is quite different from that in commercial LIBs, since the cathode degradation mechanism is different for sulfide solid electrolytes and organic liquid electrolytes. Thus, it is necessary to study cathode coating materials from a new perspective for ASSBs. Furthermore, most coating research for ASSBs has focused on the modification of LiCoO_2_ cathodes. In light of the recent drive to reduce the cobalt content of large-scale battery systems owing to its prohibitive cost, surface coatings are needed that are suitable for cathodes with low Co content.

In this work, we selected Li[Ni_0.8_Co_0.15_Al_0.05_]O_2_ (NCA), a high Ni cathode material suitable for large scale LIBs, and applied a new coating material to its surface in an attempt to enhance its interfacial stability with a sulfide solid electrolyte (75Li_2_S–22P_2_S_5_–3Li_2_SO_4_) in an all-solid-state cell. The influence of the surface coating on the electrolyte/cathode interface is highly influenced by the coating material^27–32^. Although they have not yet been applied as cathode coating materials to our knowledge, we expect indium oxides to be promising coating materials as they are highly stable^33^. Since a lithium depletion layer is formed between cathode/sulfide electrolyte interface, oxides containing lithium is preferable as coating materials for sulfide based ASSBs. Fortunately, LiInO_2_ with α-NaFeO_2_ type structure has been reported as stable oxide^34,35^. Hence, we selected LiInO_2_ as a coating material for the NCA cathode.

In addition, to investigate whether enhancing the ionic conductivity of the oxide coating layer could enhance the transfer of lithium ions at the cathode/electrolyte interface, iodine ions were introduced to form a LiInO_2_–LiI composite coating material. Although surface coating can suppress unwanted reactions at the cathode/electrolyte interface, lithium ion transfer at the interface is still difficult. Thus, providing the coating material with good ionic conductivity is expected to improve the movement of lithium ions at the interface. Lithium halides (such as LiI), have been effectively utilized to increase the conductivity of oxides and sulfides^36–40^. If LiI in the LiInO_2_ coating layer reacts with the sulfide electrolyte, it could form a highly conductive interfacial layer.

In this study, the electrochemical properties of LiInO_2_- and LiInO_2_–LiI-coated NCA electrodes were characterized and compared those of a pristine electrode. Scanning transmission electron spectroscopy (STEM), energy dispersive X-ray spectroscopy (EDS), electron energy loss spectroscopy (EELS), and X-ray photoelectron spectroscopy (XPS) were performed to analyse the effect of the coating materials.

### LiInO_2_-coated NCA cathode

Various amounts of LiInO_2_ (0.5–2 wt.%) were applied to NCA powder as a coating material. Figure S1 shows the surface morphologies of the pristine and LiInO_2_-coated NCA powders observed by SEM. The overall shape of the powder was not changed distinctly by the surface coating. However, the coated powder was covered with nano-sized particles, which are expected to be LiInO_2_ particles. The coverage of this coating layer increased with the amount of LiInO_2_ added. To determine the uniformity of the coating layer, the surface of the 1 wt.% LiInO_2_-coated NCA powder was analysed using TEM-EDS. Figure S2 presents an elemental map of the 1 wt.% coated powder. Ni, Co, O, and In were all distributed uniformly on the surface of the NCA powder, indicating that the LiInO_2_ coating layer was well dispersed on the surface.

XRD patterns of the pristine and LiInO_2_-coated NCA powder were investigated to compare the phase integrity of the powder before and after coating. As shown in Fig. S3, all the diffraction peaks of the pristine and LiInO_2_-coated powders matched the general patterns of the α-NaFeO_2_ structure (space group *R* $\bar{3}$*m*) without notable impurity peaks, implying that the coating process did not deteriorate the phase integrity of the NCA powder.

**Figure 1:**
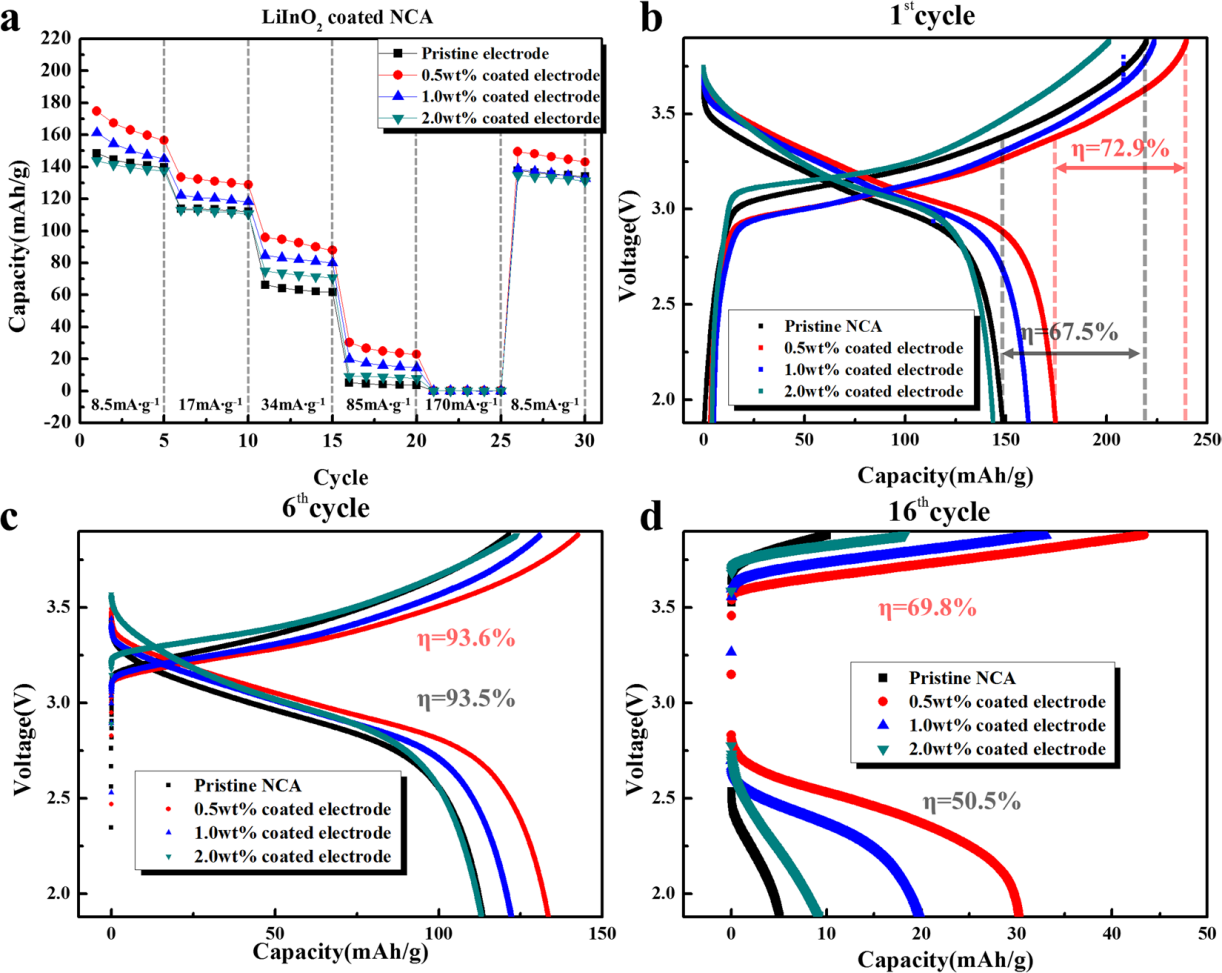
Electrochemical properties of composite electrodes. (**a**) Discharge capacities of the pristine, 0.5 wt.%, 1.0 wt.%, and 2.0 wt.% LiInO_2_-coated NCA electrodes at current densities of 8.5, 17, 34, 85, and 170 mA·g^−1^ over the voltage range of 3.88–1.88 V. Charge–discharge profiles of the pristine and LiInO_2_-coated electrodes at (**b**) 8.5 mA·g^−1^, (**c**) 17 mA·g^−1^, and (**d**) 85 mA·g^−1^.

The pristine and LiInO_2_-coated NCA powders were mixed with sulfide solid electrolyte and Super P carbon black to form composite electrodes, and all-solid-state cells were prepared to measure the electrochemical properties. Figure 1a shows the discharge capacities of the electrodes in all-solid-state cells at current densities of 8.5–170 mA·g^−1^. The upper and lower cut-off voltages are 3.88 V and 1.88 V, respectively, considering the voltage drop by the anode (a Li–In composite). The capacities of the electrodes seem to be lower than those of general cells that use liquid organic electrolyte. Specially, the capacity is seriously reduced at high current densities, which results in poor rate capability. This indicates that the internal resistance of the all-solid-state cell is much higher than that in general cells using liquid electrolyte. Interfacial reactions between the electrolyte and cathode contribute greatly to the high internal resistance of ASSBs^21–23^. As the sulfide solid electrolyte is not thermodynamically stable in the cathode potential range, direct contact between the oxide cathode and sulfide electrolyte results in the formation of a lithium-deficient space charge layer. The bonding between oxide and lithium ions is much stronger than that between sulfide and lithium ions, which leads to the transfer of lithium ions from the electrolyte to the cathode^41,42^. Secondary reactions between sulfide and oxide ions, attributed to the diffusion of transition metal ions (S and P) from the sulfide electrolyte (75Li_2_S–22P_2_S_5_–3Li_2_SO_4_), also form an undesirable reaction layer. As shown in Fig. 1a, the LiInO_2_-coated electrodes presented somewhat higher discharge capacities than the pristine electrode. This may because the LiInO_2_ coating suppresses undesirable reactions between the sulfide electrolyte and oxide cathode, as we predicted. The discharge capacities, capacity retentions, and coulombic efficiency of the pristine and coated electrodes are summarized in Table 1.

**Table 1:** Properties of pristine and LiInO_2_-coated electrode at different current densities.

Current density	Pristine electrode			0.5 wt.% coated electrode			1 wt.% coated electrode			2 wt.% coated electrode		
	DC^*^ (mAh·g^−1^)	CR^†^ (%)	η^‡^ (%)	DC (mAh·g^−1^)	CR (%)	η (%)	DC (mAh·g^−1^)	CR (%)	η (%)	DC (mAh·g^−1^)	CR (%)	η (%)
8.5 mAh·g^−1^	148.6	100^§^	67.5	174.7	100	72.9	161.27	100	72.1	143.7	100	71.4
17 mAh·g^−1^	113.6	76.4	93.5	133.5	76.4	93.6	122.3	75.8	93.4	113.0	78.6	91.2
85 mAh·g^−1^	5.1	3.4	50.5	30.2	17.3	69.8	19.8	12.2	59.7	9.1	6.3	49.9

^*^DC—discharge capacity; ^†^CR—capacity retention; ^‡^η—coulombic efficiency (%). ^§^The capacity retention refers to the percentage of retained capacity at each current density compared to that at 8.5 mA·g^−1^.

Figure 1b–d show charge–discharge profiles of the pristine and coated electrodes. The 0.5 wt.% coated electrode showed a discharge capacity of about 175 mAh·g^−1^ at a current density of 8.5 mA·g^−1^. In contrast, the discharge capacity of the pristine electrode was just ~149 mAh·g^−1^ at the same current density. The coulombic efficiency of the all-solid-state cells during the first cycle was significantly lower than that of general cells using liquid electrolyte (at least 90~95%). However, the LiInO_2_ coating was observed to increase the columbic efficiency. At higher current densities (17 and 85 mA·g^−1^), the LiInO_2_-coated electrodes presented superior capacity compared to the pristine electrode. The optimum coating amount within our experimental condition was 0.5 wt.%, as increasing the coating amount resulted in a decrease of capacity. This may imply that a large amount of surface coating can disturb the movement of ions and electrons in the composite cathode.

Figure S4 shows the cyclic performances of the electrodes at a current density of 17 mA·g^−1^. The all-solid-state cells all exhibited stable operation. The discharge capacity of the cells slowly decreased over the duration of the test, although no serious capacity fading was observed within our experimental conditions. This capacity fading seen here seems to be attributed to degradation of the mechanical contact between the cathode and solid electrolyte^22^, as well as to the undesirable side reactions at the cathode, electrolyte, and interface layer during cycling. For example, volume expansion/contraction of the oxide cathode material during cycling can easily deteriorate the contact between the cathode and electrolyte. This problem remains a challenging task for the commercialization of all-solid-state cells. As can be expected from Fig. 1a,c, the LiInO_2_-coated electrodes exhibited higher discharge capacities than the pristine electrode, highlighting the beneficial effect of the coating.

**Figure 2:**
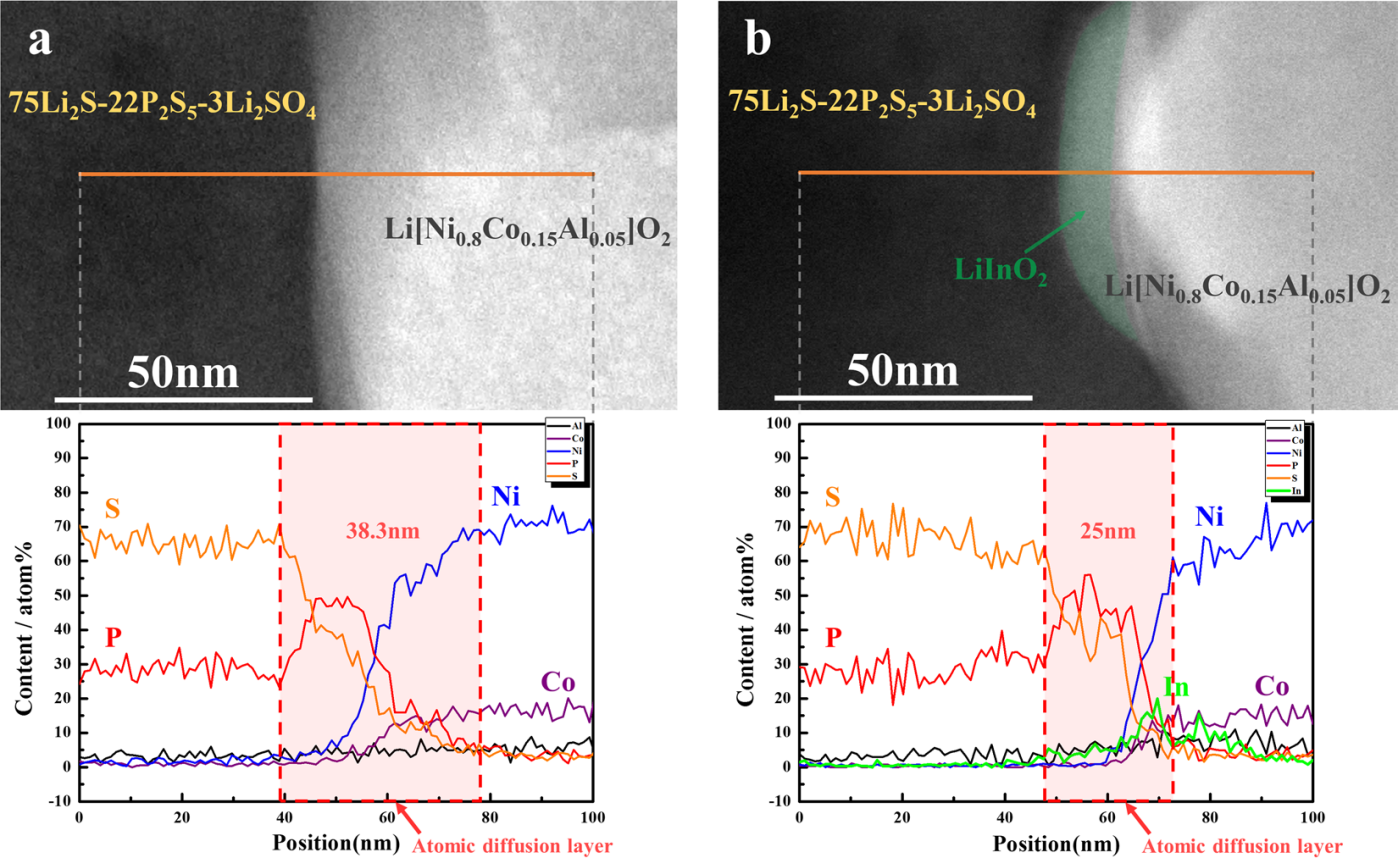
Cross-sectional HAADF-STEM image and EDS line profiles for Ni, Co, In, P, and S at the electrolyte/cathode interface (75Li_2_S–22P_2_S_5_–3Li_2_SO_4_/NCA) after 30 charge–discharge cycles. (**a**) Pristine and (**b**) LiInO_2_-coated NCA.

To validate the suppression of the secondary reaction by surface modification, the pristine and LiInO_2_-coated (0.5 wt.%) NCA electrodes were investigated by STEM-EDS after first being subjected to 30 charge–discharge cycles at a current density of 17 mA·g^−1^. Figure 2 presents the cross-sectional STEM images and EDS line profiles of the interface between the sulfide solid electrolyte (75Li_2_S–22P_2_S_5_–3Li_2_SO_4_) and the pristine or coated NCA cathode (Li[Ni_0.8_Co_0.15_Al_0.05_]O_2_). With the pristine cathode, a considerably thick (~38 nm) interfacial reaction layer was observed at the cathode/electrolyte interface (Fig. 2a). Significant amounts of S and P had penetrated the oxide cathode, which clearly shows the interfacial instability.

In the coated sample, the existence of a LiInO_2_ layer was confirmed by STEM and EDS, as shown in Fig. 2b (marked in green). Interestingly, the diffusion of S and P was considerably suppressed by the LiInO_2_ layer. From the EDS line profile of the interface, it appears as if their penetration was stopped by the presence of In. The interfacial reaction layer was also somewhat decreased by the LiInO_2_ coating. These results indicate that the unwanted side reactions at the cathode/electrolyte interface were suppressed by coating the NCA cathode material with LiInO_2_. The LiInO_2_ layer, which is more stable than the cathode material, prevents direct contact between the sulfide electrolyte and the cathode, thereby suppressing unwanted side reactions. The enhanced rate capability and increased capacity of the LiInO_2_-coated cathode can be explained by this effect. However, the undesirable interface layer was still formed, meaning that the movement of lithium ions between the cathode and electrolyte is deteriorated despite the LiInO_2_ coating.

### LiInO_2_-LiI-coate NCA cathode

To promote lithium-ions transfer across the cathode/electrolyte interfacial layer, LiI was incorporated into the LiInO_2_ coating material. The addition of I ions is expected to enhance the ionic conductivity of the LiInO_2_ coating layer, which may improve the rate capability of the all-solid-state cells. Before being applied as a coating, LiInO_2_ was mixed with LiI and heat treated to observe the phase change of LiInO_2_ with different LiI additions. As shown in the XRD pattern in Fig. S5, when 0.25 mol% of LiI was added, the diffraction peaks of the LiInO_2_–LiI composite were not significantly different to that of LiInO_2_, except for a slight decrease in peak intensity. However, when 1 mol% LiI was added, several peaks were newly formed in the diffraction patterns, which means the phase change of the LiInO_2_ or formation of secondary phase. Since we wanted to use I ions as doping material for LiInO_2_, the amount of LiI was adjusted to 0.25 mol% of LiInO_2_. In practice, LiInO_2_ will react with not only Li and I but also transition metals or impurities such as LiCO_3_ from the cathode surface. Therefore, the exact composition of the coating layer is difficult to predict. For convenience, a 0.5 wt.% LiInO_2_ coating material to the cathode containing 0.25 mol% LiI is hereafter denoted as a ‘LiInO_2_–LiI coating’.

Figure S6a presents a cross-sectional STEM image of a composite electrode composed of sulfide electrolyte (75Li_2_S–22P_2_S_5_–3Li_2_SO_4_) and LiInO_2_–LiI-coated cathode. The coating layer seems to have been preserved at the cathode/electrolyte interface. To observe the composition of the coating layer in more detail, the composite electrode was studied by EDS. As shown in Fig. S6b, I was detected in the interface layer along with In, which confirms the formation of a LiInO_2_–LiI composite layer.

**Figure 3:**
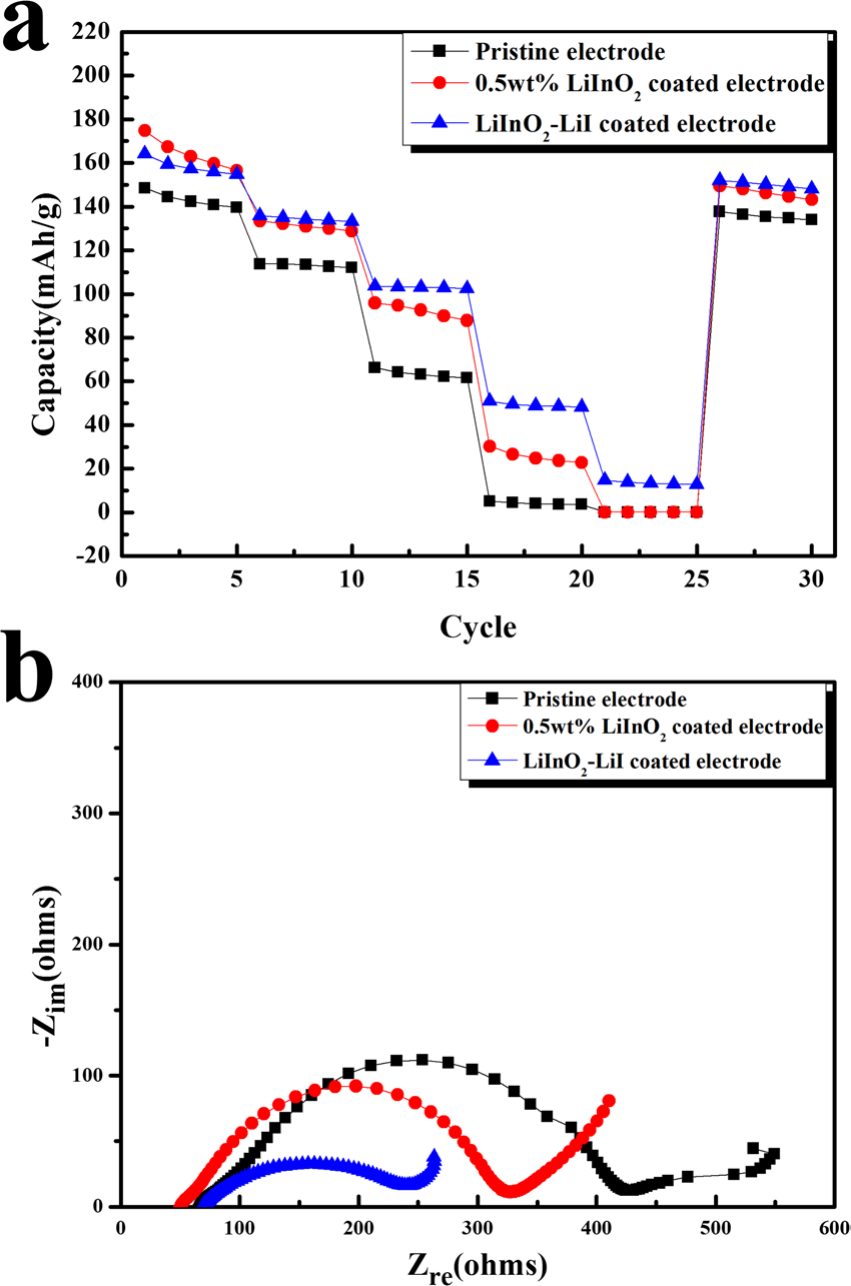
Electrochemical properties of composite electrodes. (**a**) Discharge capacities of the pristine, 0.5 wt.% LiInO_2_-coated, and 0.5 wt.% LiInO_2_–LiI-coated electrodes in all-solid-state cells at current densities of 8.5, 17, 34, 85, and 170 mA·g^−1^. (**b**) Nyquist plots of the pristine, LiInO_2_-coated, and LiInO_2_–LiI-coated electrodes in all-solid-state cells.

Figure 3a shows the discharge capacity of the pristine, 0.5 wt.% LiInO_2_-coated, and 0.5 wt.% LiInO_2_–LiI-coated electrodes in all-solid-state cells at current densities of 8.5–170 mA·g^−1^. The initial capacity of the LiInO_2_–LiI-coated electrode was slightly smaller than that of the LiInO_2_-coated electrode. However, as the current density increased, the LiInO_2_–LiI-coated electrode exhibited a superior capacity to the LiInO_2_-coated electrode. This means that adding LiI to the coating layer can successfully enhance the rate capability of all-solid-state cells with coated cathodes. Figure 3b presents Nyquist plots of the pristine, LiInO_2_-coated, and LiInO_2_–LiI-coated electrodes in all-solid-state cells. All the Nyquist plots were composed of 2–3 overlapped semicircles. The typical Nyquist plots of lithium ion cells (using liquid electrolyte) consist of two semicircles, attributed to charge transfer resistance and the solid electrolyte interface. However, for cells using a sulfide solid electrolyte, the impedance is expected to be highly dependent upon resistance related to the interface layer between the oxide cathode and the sulfide electrolyte. The Nyquist plot of the pristine electrode shows a large semicircle followed by a small semicircle at low frequency region. In contrast, the Nyquist plots of the coated samples presented a distorted semicircle, which may consist of overlapped 2–3 semicircles. It is notable that the semicircles of the coated electrode were decreased compared to that of the pristine electrode. In particular, the Nyquist plot of the LiInO_2_–LiI-coated electrode showed a significantly smaller semicircle than not only the pristine electrode but also the LiInO_2_-coated electrodes. This results indirectly proves that the addition of I ions is efficient to reduce the impedance of the cells and enhance the lithium ion transfer, which leads to the fast rate capability of the LiInO_2_–LiI-coated electrode shown in Fig. 3a.

**Figure 4:**
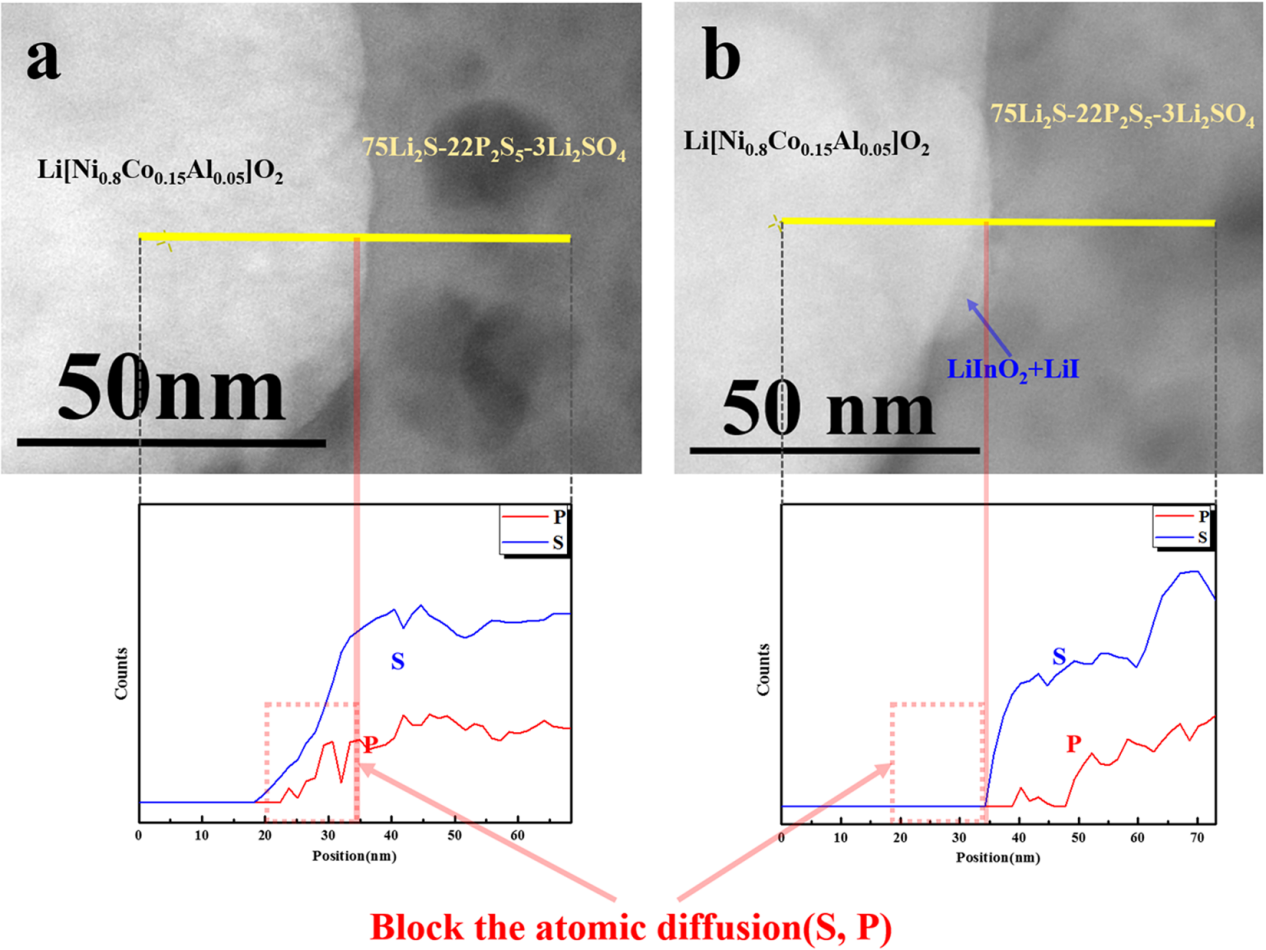
Cross-sectional STEM images and EELS line profiles of electrolyte/cathode interface (75Li_2_S–22P_2_S_5_–3Li_2_SO_4_/NCA) after 30 charge–discharge cycles. (**a**) Pristine and (**b**) LiInO_2_–LiI-coated NCA.

Figure 4 presents cross-sectional STEM images and EELS line profiles of cathode/electrolyte interface of the pristine and 0.5 wt.% LiInO_2_–LiI-coated electrodes that were collected from the composite electrode of all-solid-state cells after 30 charge–discharge cycles. In the pristine sample, S and P ions were detected in the cathode region, as shown in Fig. 4a, indicating that a considerable number of ions from the sulfide electrolyte penetrated the cathode. However, as shown in Fig. 4b, the S and P ions almost entirely disappeared from the cathode region with the LiInO_2_–LiI coating layer. This clearly confirms that the LiInO_2_–LiI coating layer successfully blocks the diffusion of the S and P ions from the sulfide electrolyte to the cathode.

**Figure 5:**
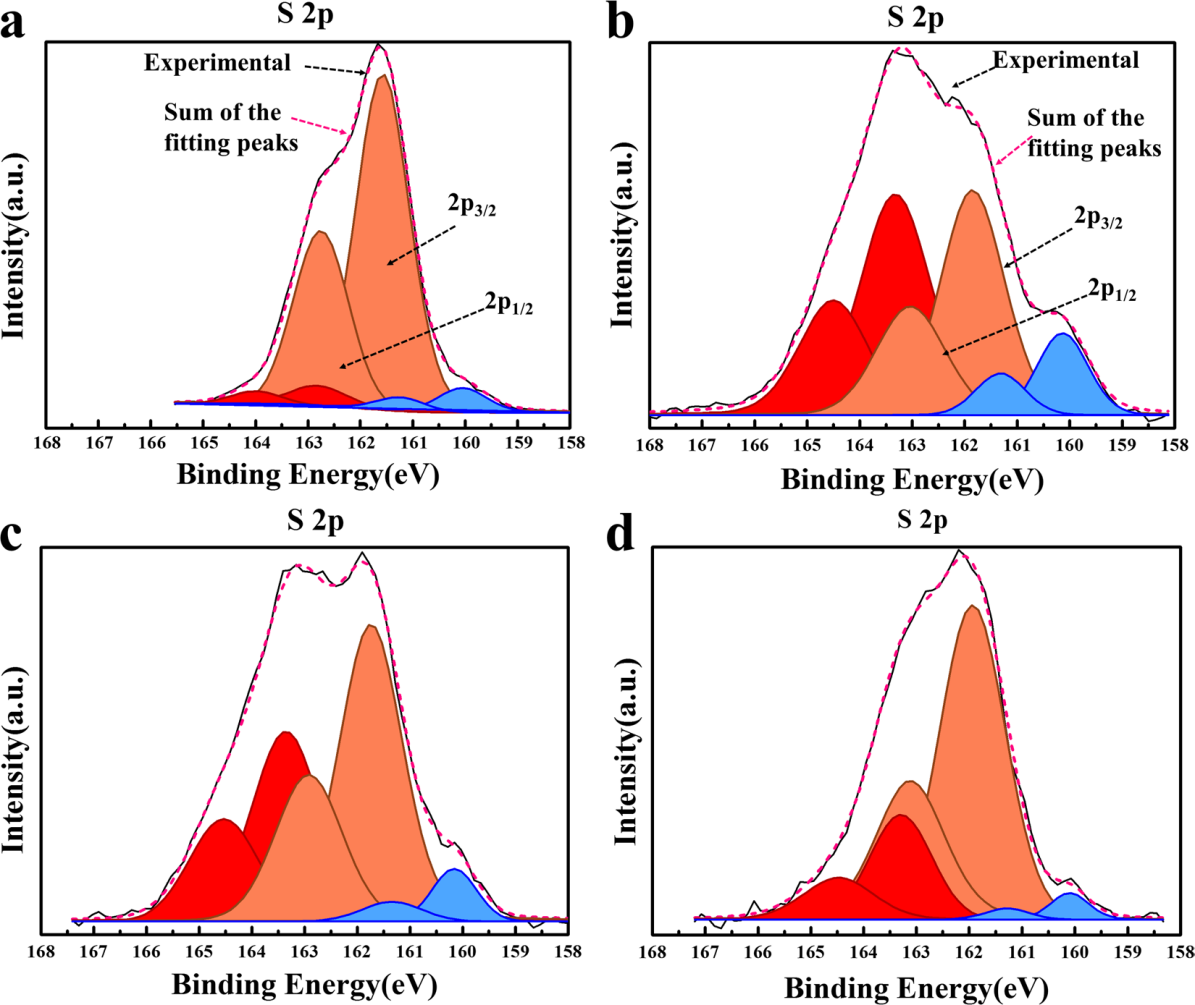
S 2p XPS spectra of the pristine electrolyte and composite electrodes of the all-solid-state cells. (**a**) 75Li_2_S–22P_2_S_5_–3Li_2_SO_4_ solid electrolyte (pristine). (**b**–**d**) Composite electrodes after 30 charge–discharge cycles employing (**b**) pristine, (**c**) LiInO_2_-coated, and (**d**) LiInO_2_–LiI-coated cathodes.

To determine the effect of the LiI addition to the coating layer in detail, composite electrodes employing pristine, 0.5 wt.% LiInO_2_-coated, and 0.5 wt.% LiInO_2_–LiI-coated cathodes were compared using XPS analysis (Fig. 5). The composite electrodes were collected from all-solid-state cells that had been subjected to 30 charge–discharge cycles. We first analysed the solid electrolyte (75Li_2_S–22P_2_S_5_–3Li_2_SO_4_) before the cathode was added. As shown in Fig. 5a, the S 2p spectrum of the pristine sulfide electrolyte was composed of two main peaks at ~161.5 eV and ~162.8 eV (marked in orange). These peaks are attributed to the S 2p_1/2_ and 2p_3/2_ components of non-bridging sulfur (S^−^) in the sulfide electrolyte^43^. The small peaks marked in red almost correspond to the peaks of binding sulfur (S^0^) in P_2_S_7_^4−^ units. However, we cannot exclude the possibility of these peaks arising from the products of secondary reactions such as the oxidation of sulfide. The peaks marked in blue are likely to be associated with the side reaction as well.

The XPS spectra of the coated-cathode composite electrodes (after 30 cycles) presented more complicated peaks. As shown in Fig. 5b–d, the 2S XPS spectrum of the composite electrode employing pristine cathode still showed two main peaks at ~161.5 eV and ~162.8 eV (marked in orange). However, their intensities were somewhat reduced. Instead, the peaks at ~163.3 eV and ~164.5 eV (marked in red) and the peaks at ~160.1 eV and ~161.4 eV (marked in blue) were significantly increased. The growth of these peaks is related to the secondary reaction between the cathode and sulfide electrolyte. Interestingly, the peaks attributed to the side reaction were considerably decreased when the LiInO_2_ coating was applied, as shown in Fig. 5c. Moreover, the intensity of the main peaks, attributed to non-bridging sulfur (S^−^) (marked in orange), were somewhat higher due to the LiInO_2_ coating effect. This result coincides with the previous STEM-EDS analysis (Fig. 2), indicating that LiInO_2_ coating is an effective method to suppress secondary reactions between the sulfide electrode and oxide cathode.

Notably, the side reaction seemed to be even more suppressed by adding LiI to the LiInO_2_ coating layer (Fig. 5d). The 2S XPS peaks thought to be related to the secondary reactions (marked in red and blue) were significantly reduced compared with those in the composite electrodes with both pristine and LiInO_2_-coated cathodes. This shows that the LiInO_2_–LiI composite coating is more effective than the LiInO_2_ coating to suppress the unwanted reactions between the electrolyte and cathode.

The main purpose of adding LiI to the LiInO_2_ was to enhance the conductivity of the coating layer and increase the rate capability of all-solid-state cells. As shown in Fig. 3, the LiInO_2_–LiI-coated electrode presented a higher rate capability and lower impedance than the LiInO_2_-coated electrode. In addition, it was confirmed that the undesirable reactions at the sulfide electrolyte/cathode interface were suppressed by this coating, which seemed to mean that the LiInO_2_–LiI coating layer is less reactive with sulfide electrolyte than LiInO_2_ coating. The superior rate capability of the LiInO_2_–LiI-coated electrode is associated with this enhanced protection effect as well as the improved conductivity due to I doping.

## Summary

**Figure 6:**
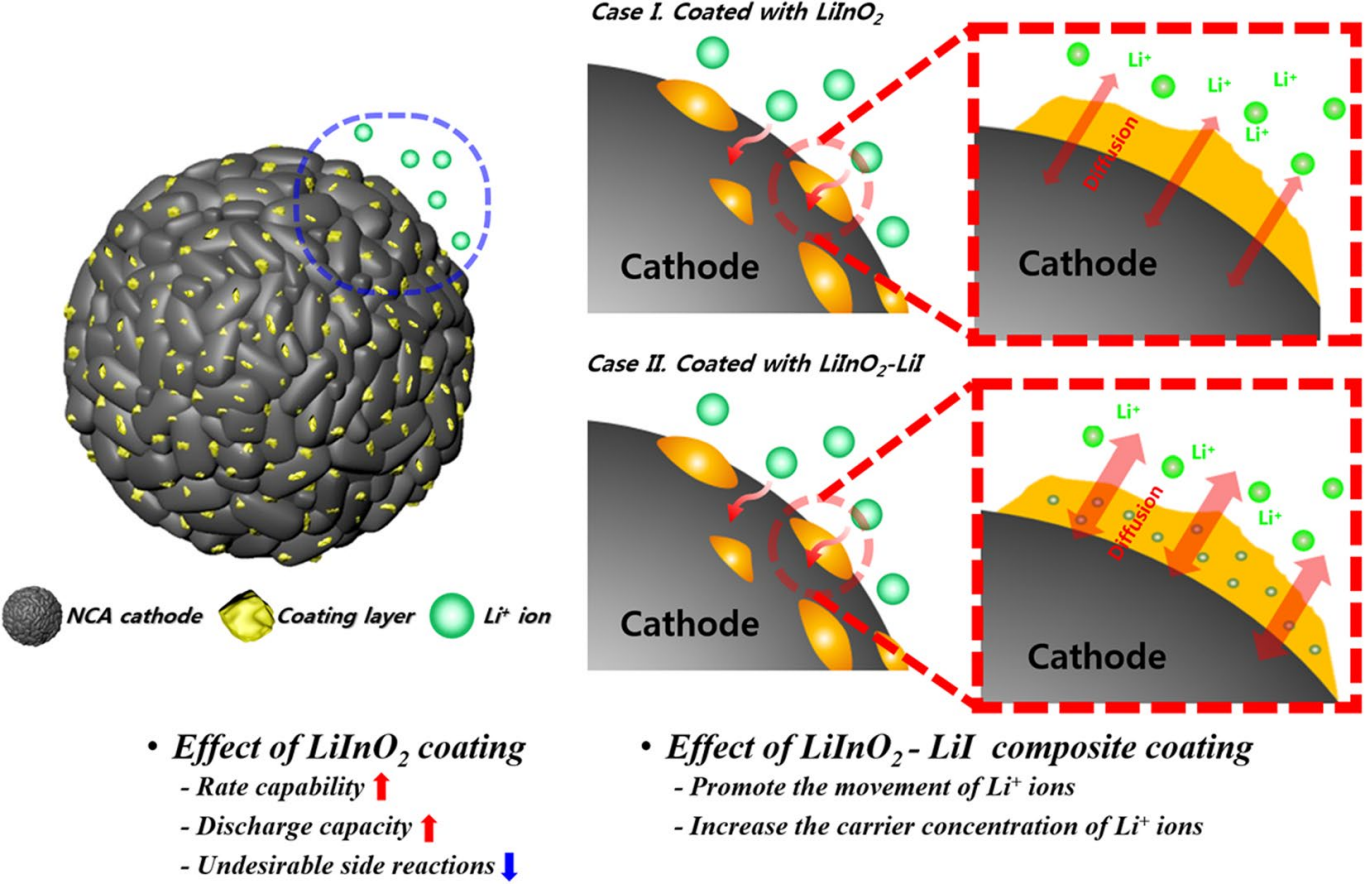
Schematic diagram showing the effect of LiInO_2_ and LiInO_2_–LiI coating on the surface of the NCA cathode.

To increase the interfacial stability between the sulfide electrolyte (75Li_2_S–22P_2_S_5_–3Li_2_SO_4_) and oxide cathode (NCA) in all-solid-state cells, LiInO_2_ and LiInO_2_–LiI were employed as new cathode coating materials. The coating layer on the surface of the cathode powder comprised homogeneously distributed nano-sized LiInO_2_ particles. The stability of the coated cathodes was evaluated by fabricating sulfide-based all-solid-state cells. The composite electrode employing LiInO_2_-coated NCA showed superior capacity and rate capability to the composite electrode employing pristine cathode, and that employing LiInO_2_–LiI-coated NCA showed better rate capability and lower impedance compared to both other types of electrode. Cross-sectional STEM images and EDS line profiles of the sulfide electrolyte/cathode interfaces demonstrated that the LiInO_2_ coating layer suppressed the diffusion of S and P ions into the cathode, indicating that the coating reduced the undesirable interfacial reactions. This phenomenon was more pronounced with the LiInO_2_–LiI coating material. In the STEM-EELS analysis, it was observed that the LiInO_2_–LiI coating layer effectively prevented the diffusion of S and P ions into the cathode region. XPS analyses confirmed that the LiInO_2_–LiI coating layer was better at suppressing the secondary reactions at the sulfide electrolyte/cathode interface than the LiInO_2_ coating. Hence, surface modification by coating with LiInO_2_–LiI is a promising method of reducing interfacial instability and enhancing the electrochemical properties of sulfide-based all-solid-state cells. Figure 6 summarizes the protective effect of the LiInO_2_ and LiInO_2_–LiI coatings on the surface of the NCA cathode.

## Methods

### Materials and coating procedure

A commercial Li[Ni_0.8_Co_0.15_Al_0.05_]O_2_ (NCA) powder was used as a pristine cathode. To prepare the LiInO_2_ coating solution, lithium nitrate (LiNO_3_, Aldrich) and indium nitrate hydrate (In(NO_3_)_3_∙*x*H_2_O) were dissolved in anhydrous ethanol (99.9%, Aldrich) at 70 °C. The amount of LiInO_2_ was adjusted to 0.5 wt.%, 1.0 wt.%, and 2.0 wt.% of the cathode powder. For the LiInO_2_–LiI coating solution, ammonium iodide and lithium nitrate were also dissolved in the solution of LiInO_2_ in anhydrous ethanol, at 0.25 mol% or 1 mol% relative to the molar ratio of LiInO_2_. Pristine cathode powder was added to the coating solution and stirred at 70 °C until the solvent was completely evaporated. The dried materials were heat treated at 650 °C (ramping rate = 2 °C/min.) for 5 h under air atmosphere to obtain LiInO_2_-coated NCA powder. As samples for comparison, the LiInO_2_–LiI coating solutions were dried at 70 °C and heat treated at 650 °C for 5 h, which is same condition for the coating process, to obtain the LiInO_2_–LiI composite powders.

### Sample characterization

X-ray diffraction (XRD) patterns of synthesized samples of pristine and LiInO_2_-coated NCA powders as well as LiInO_2_ were obtained using a Rigaku MiniFlex II X-ray diffractometer over the 2θ range of 10–90° with monochromatized Cu Kα radiation (λ = 1.5406 Å). The surface morphology of the pristine and coated powder was observed using field-emission scanning electron microscopy (FE-SEM, Nova Nano 200) and high-resolution transmission electron microscopy (HR-TEM, JEOL JEM-2100F).

### All-solid-state cell fabrication

For electrochemical testing, all-solid-state cells were fabricated using sulfide electrolyte (75Li_2_S–22P_2_S_5_–3Li_2_SO_4_). The sulfide electrolyte was prepared using mechanical milling process and subsequent heat-treatment, as reported in the previous literature^44^. The cathode mixture for the composite electrode was prepared by hand mixing the cathode (pristine or coated NCA), sulfide solid electrolyte, and Super P carbon black at a weight ratio of 68.6: 29.4: 2.0. To form a solid electrolyte layer as a separator, 0.2 g of the sulfide electrolyte was compressed under 30 MPa pressure in a mould. Thereafter, the composite electrode (cathode) layer was formed on one side of the solid electrolyte using 0.02 g of the cathode mixture and carbon-nanotube paper (Hanwha chemical). The anode electrode layer was formed on the opposite side with 0.05 g of Li–In powder and nickel foil. Each compression process was performed at 30 MPa. The cathode/electrolyte/anode assembly was placed inside a 2032 coin-type cell. The structure of this assembly is shown in Fig. S7.

### Electrochemical properties

The cells were subjected to galvanostatic cycling (WonATech voltammetry system) over a voltage range of 3.88–1.88 V at various charge–discharge rates. X-ray photoelectron spectroscopy (XPS, Thermo Scientific K-Alpha) was employed to analyse the reaction products on the composite electrodes containing pristine, LiInO_2_-coated, and LiInO_2_–LiI-coated NCA powders. The all-solid-state cells were cycled 30 times, then the composite electrodes were separated from the cells and stored in a dry box. The electrodes were held under vacuum while being transferred to the instruments, and etched by ~200 nm using Ar sputtering to remove contamination on the surface before analysis. Scanning transmission electron microscopy (STEM; Titan 80-300), energy dispersive X-ray spectroscopy (EDS), and electron energy loss spectroscopy (EELS) analyses were employed to investigate the element diffusion between the cathode and the solid electrolyte. The cross-sectional interface was prepared using focused ion beam (FIB) milling (Quanta 3D FEG) prior to STEM analyses. A vacuum transfer system was utilized to avoid exposure to air during the FIB milling process and transfer to the STEM.

## Supplementary information

Supporting Information
