# Supplementary material for: Cathode coating using LiInO2-LiI composite for stable sulfide-based all-solid-state batteries
Source: Sci Rep. 2019 May 30;9:8099. doi: 10.1038/s41598-019-44629-x (PMC6543035; doi:10.1038/s41598-019-44629-x)
Supplement: Supplementary file 1 — Supporting Information [file 41598_2019_44629_MOESM1_ESM.pdf]

Cathode coating using $LiInO_2$-LiI composite for stable sulfide-based all-solid-state batteries

Hwan Wook Kwak and Yong Joon Park*

Department of Advanced Materials Engineering, Kyonggi University, 154-42, Gwanggyosan-Ro, Yeongtong-Gu, Suwon-Si, Gyeonggi-Do, 16227, Republic of Korea

*Corresponding author

Ph: +82-31-249-9769; E-mail: yjpark2006@kyonggi.ac.kr

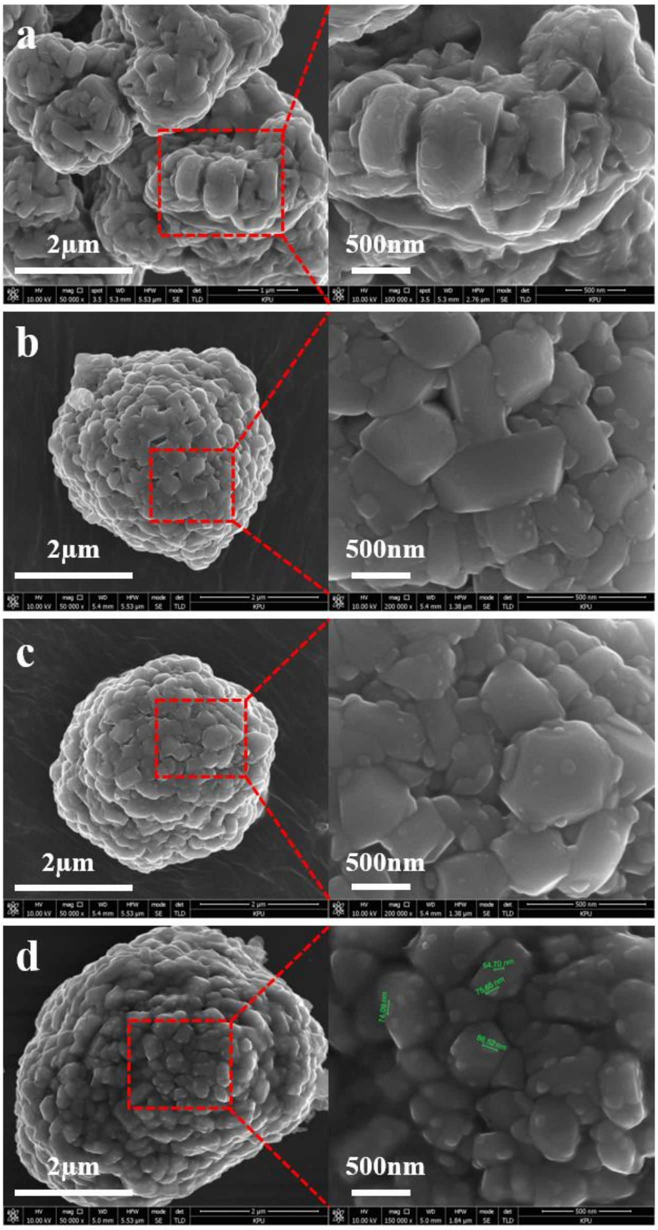

Figure S1 | SEM images of pristine and coated NCA powder. (a) Pristine, (b) 0.5 wt.% $LiInO_2$-coated, (c) 1.0 wt.% $LiInO_2$-coated, and (d) 2.0 wt.% $LiInO_2$-coated powders.

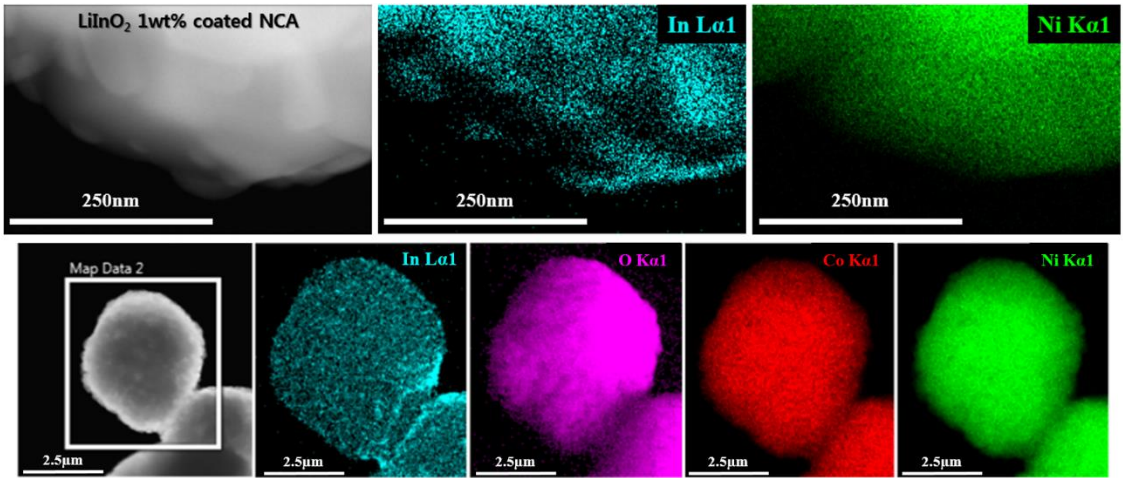

Figure S2 | TEM and elemental mapping images of the 1.0 wt.% $LiInO_2$-coated NCA powder.

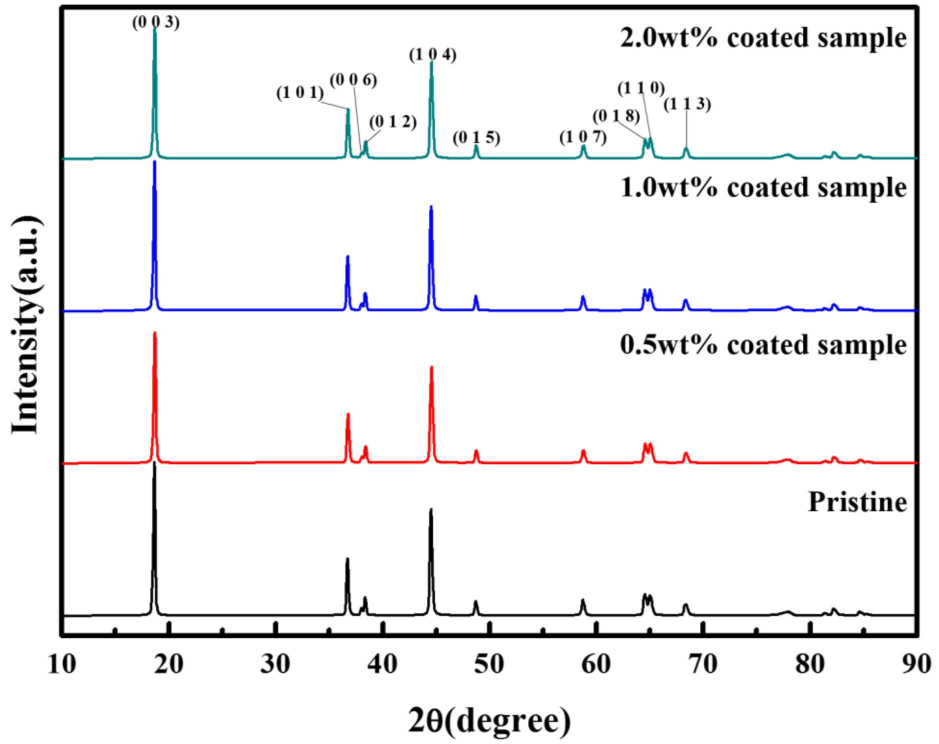

Figure S3 | XRD patterns of pristine and $LiInO_2$-coated NCA powders.

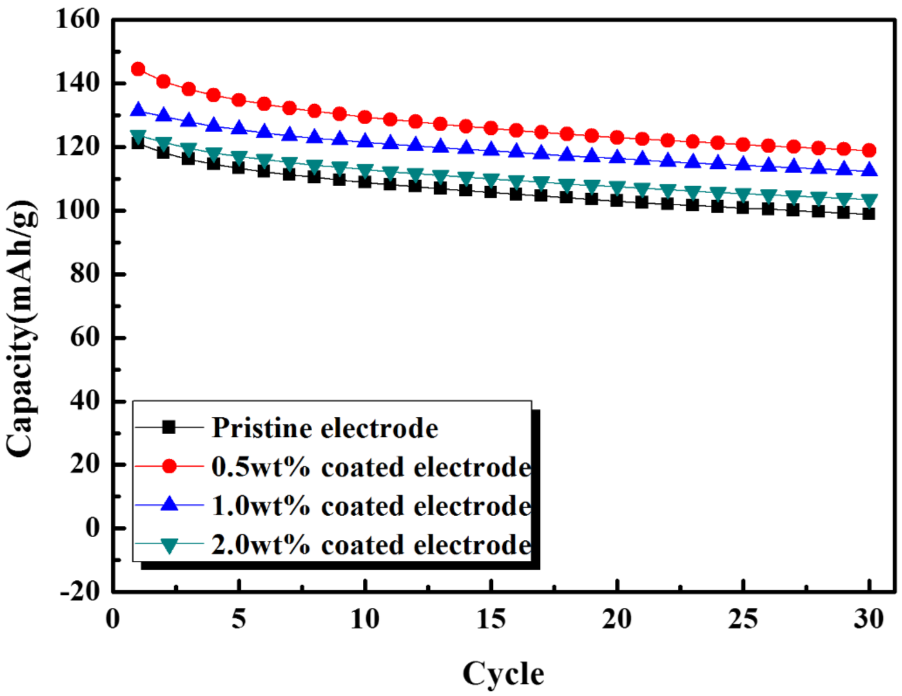

Figure S4 | Cyclic performance of the pristine, 0.5 wt.%, 1.0 wt.%, and 2.0 wt.% $LiInO_2$-coated NCA electrodes at a current density of 17 $mA \cdot g^{-1}$.

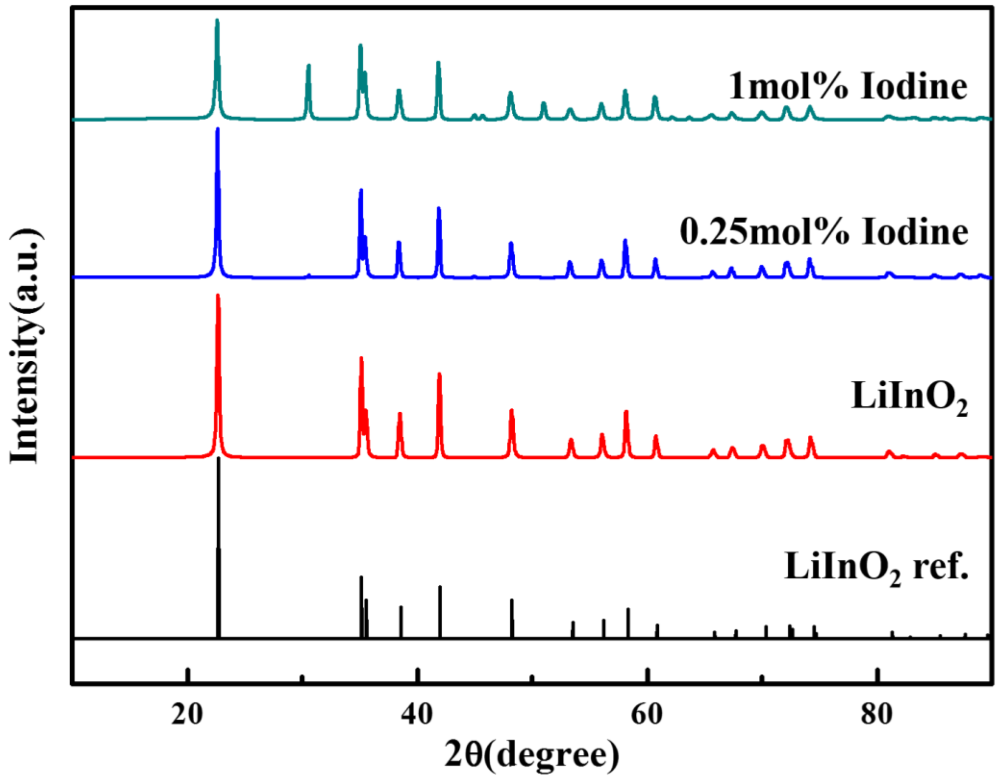

Figure S5 | XRD patterns of $LiInO_2$ and $LiInO_2$–LiI composites.

.

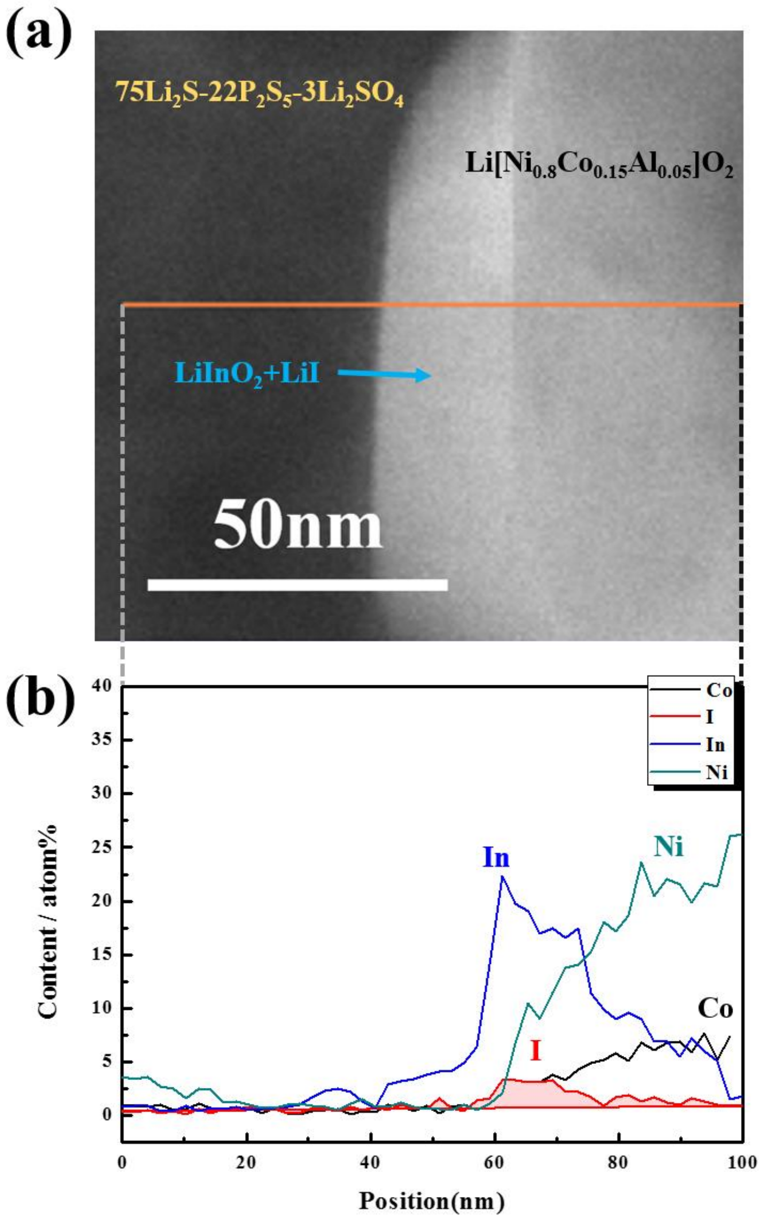

Figure S6 | (a) Cross-sectional STEM image and (b) EDS line profile of the composite electrode composed of sulfide electrolyte ($75Li_2S–22P_2S_5–3Li_2SO_4$) and $LiInO_2$–LiI-coated cathode.

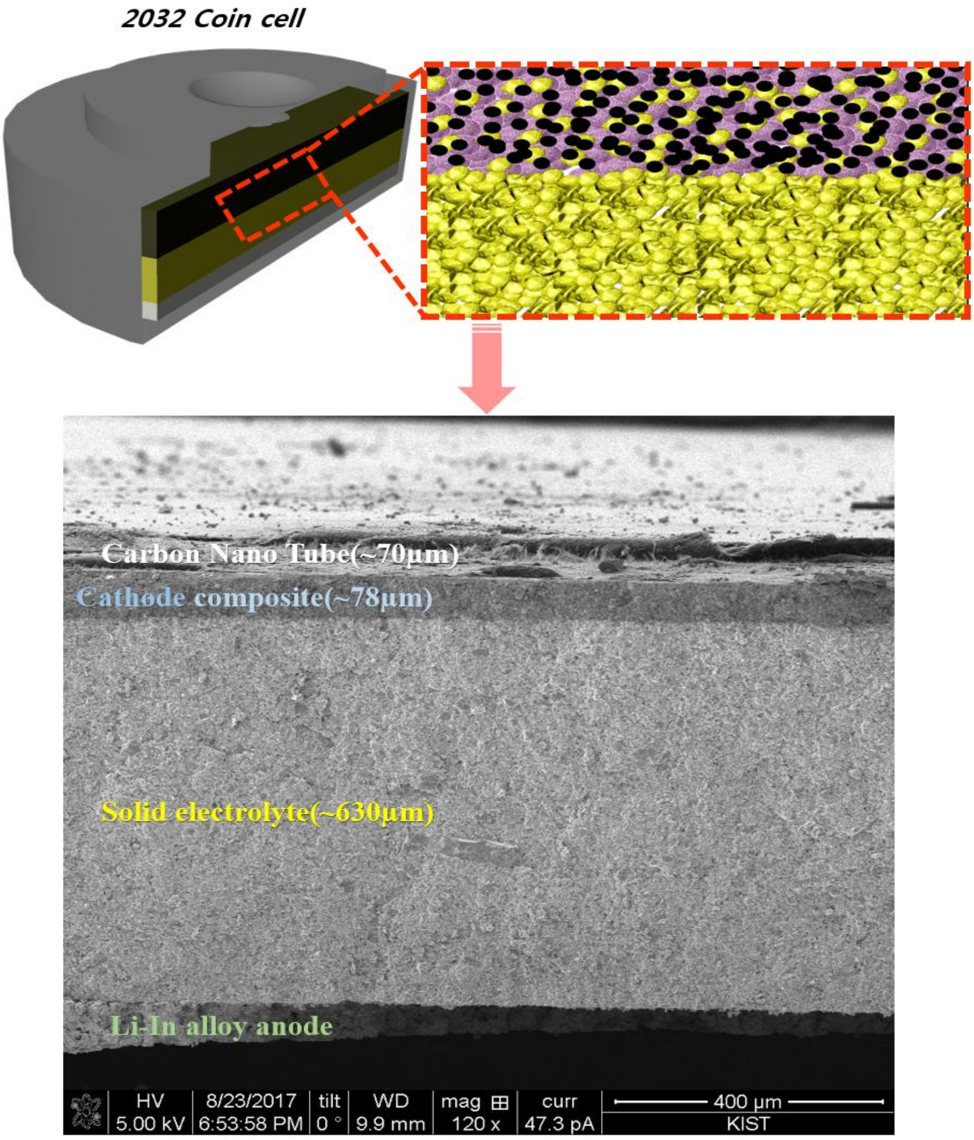

Figure S7 | Schematic illustration demonstrating the structure of all-solid-state cells comprising composite cathode and sulfide electrolyte.
